# Gluconeogenesis in the extraembryonic yolk syncytial layer of the zebrafish embryo

**DOI:** 10.1093/pnasnexus/pgae125

**Published:** 2024-03-21

**Authors:** Fumiya Furukawa, Akihiro Aoyagi, Kaori Sano, Keita Sameshima, Miku Goto, Yung-Che Tseng, Daisuke Ikeda, Ching-Chun Lin, Katsuhisa Uchida, Sei-ichi Okumura, Ko Yasumoto, Mitsuru Jimbo, Pung-Pung Hwang

**Affiliations:** School of Marine Biosciences, Kitasato University, 1-15-1 Kitazato, Minami-ku, Sagamihara, Kanagawa 252-0373, Japan; Institute of Cellular and Organismic Biology, Academia Sinica, No. 128, Sec. 2, Nankang, Taipei 11529, Taiwan ROC; School of Marine Biosciences, Kitasato University, 1-15-1 Kitazato, Minami-ku, Sagamihara, Kanagawa 252-0373, Japan; Department of Chemistry, Faculty of Science, Josai University, 1-1 Keyakidai, Sakado, Saitama 350-0295, Japan; School of Marine Biosciences, Kitasato University, 1-15-1 Kitazato, Minami-ku, Sagamihara, Kanagawa 252-0373, Japan; School of Marine Biosciences, Kitasato University, 1-15-1 Kitazato, Minami-ku, Sagamihara, Kanagawa 252-0373, Japan; Institute of Cellular and Organismic Biology, Academia Sinica, No. 128, Sec. 2, Nankang, Taipei 11529, Taiwan ROC; School of Marine Biosciences, Kitasato University, 1-15-1 Kitazato, Minami-ku, Sagamihara, Kanagawa 252-0373, Japan; Institute of Cellular and Organismic Biology, Academia Sinica, No. 128, Sec. 2, Nankang, Taipei 11529, Taiwan ROC; Department of Marine Biology and Environmental Sciences, Faculty of Agriculture, University of Miyazaki, 1-1 Gakuen Kibanadai-Nishi, Miyazaki 889-2192, Japan; School of Marine Biosciences, Kitasato University, 1-15-1 Kitazato, Minami-ku, Sagamihara, Kanagawa 252-0373, Japan; School of Marine Biosciences, Kitasato University, 1-15-1 Kitazato, Minami-ku, Sagamihara, Kanagawa 252-0373, Japan; School of Marine Biosciences, Kitasato University, 1-15-1 Kitazato, Minami-ku, Sagamihara, Kanagawa 252-0373, Japan; Institute of Cellular and Organismic Biology, Academia Sinica, No. 128, Sec. 2, Nankang, Taipei 11529, Taiwan ROC

**Keywords:** development, yolk syncytial layer, gluconeogenesis, PEPCK, zebrafish

## Abstract

Yolk-consuming (lecithotrophic) embryos of oviparous animals, such as those of fish, need to make do with the maternally derived yolk. However, in many cases, yolk possesses little carbohydrates and sugars, including glucose, the essential monosaccharide. Interestingly, increases in the glucose content were found in embryos of some teleost fishes; however, the origin of this glucose has been unknown. Unveiling new metabolic strategies in fish embryos has a potential for better aquaculture technologies. In the present study, using zebrafish, we assessed how these embryos obtain the glucose. We employed stable isotope (^13^C)-labeled substrates and injected them to the zebrafish embryos. Our liquid chromatography-mass spectrometry-based isotope tracking revealed that among all tested substrate, glutamate was most actively metabolized to produce glucose in the zebrafish embryos. Expression analysis for gluconeogenic genes found that many of these were expressed in the yolk syncytial layer (YSL), an extraembryonic tissue found in teleost fishes. Generation 0 (G0) knockout of *pck2*, a gene encoding the key enzyme for gluconeogenesis from Krebs cycle intermediates, reduced gluconeogenesis from glutamate, suggesting that this gene is responsible for gluconeogenesis from glutamate in the zebrafish embryos. These results showed that teleost YSL undergoes gluconeogenesis, likely contributing to the glucose supplementation to the embryos with limited glucose source. Since many other animal lineages lack YSL, further comparative analysis will be interesting.

**Significance Statement**

Glucose is an indispensable nutrient source for development of animals. However, in oviparous animals, the yolk contains little glucose. In some fish embryos, glucose levels were found to abruptly increase in their bodies, but its origin had been unknown. Present study revealed that the developing zebrafish produce glucose in a thin, polynucleated cellular layer, called yolk syncytial layer (YSL), from yolk-derived nutrients as the carbon source. The maternally derived yolk is often considered as “complete nutrient” for the embryos, but our findings may add a little note: but some nutrients are lacking, and the YSL does supplement them.

## Introduction

Yolk consumption is an essential event for most oviparous animal embryos. Vertebrate yolk is rich in lipid and protein (1), and previous studies focused primarily on digestion and absorption of these nutrients in developing animals (2–4). Meanwhile, levels of carbohydrates and sugars are often little in the yolk, and metabolism of these has gathered little attention. Glucose, the major monosaccharide in body fluids, takes important roles in mammalian embryogenesis, where it is catalyzed via hexosamine biosynthesis pathway (HBP) and pentose phosphate pathway (PPP) for normal development (5). In zebrafish (*Danio rerio*), a model for vertebrate development, glucose is essential for normal brain development (6, 7) and induction of hematopoietic stem cell (8). While mammalian embryos are supplied with maternal glucose, embryos of oviparous animals, such as fish, face limited glucose in the yolk.

Interestingly, in embryos of Atlantic cod (*Gadus morhua*), red seabream (*Pagrus major*), and zebrafish, glucose abruptly increases before hatching (9–11), which likely meets the developmental requirement. Also, enzymatic activities for gluconeogenesis, i.e. de novo glucose production pathway, were detected in some developing trout species (12). These facts suggest that the developing fish produce glucose for themselves. However, the liver, the primary organ for glucose production, is not developed at the above stage of the zebrafish (13). Also, the embryos of Atlantic cod and the red seabream probably lacked the liver, according to other studies in cod (14) and in the close seabream species (15), respectively. Without the liver, how the fish embryos produce glucose? A previous study showed that fluorescent glucose analog migrated from the yolk sac to the brain (6), implying the role of yolk sac in glucose handling.

In teleost fishes, the yolk is enclosed in the yolk syncytial layer (YSL), the extraembryonic tissue derived from fusion of marginal blastomeres and the yolk cell (16, 17). To date, roles of YSL have been reported in the context of morphogenesis (for review, see Ref. (18)). However, its contribution to yolk handling has been less reported, despite the direct contact with the yolk. Previous studies reported ultrastructure (19) and glucose transporter gene expression (20) in YSL, but knowledge regarding metabolic role of this tissue is still limited. The lipid processing activity found in the “yolk cell” of the zebrafish embryo (3, 4) is most likely achieved by the YSL, although this has not been demonstrated. Although YSL and YSL-like tissue were found in chick (21) and sharks (22), respectively, their roles are also largely unknown.

In aquaculture fields, successful development of artificially fertilized eggs to juveniles, or seedling production, is one of the most important steps for complete culture of fish and sustainable aquaculture (23). However, the detailed understanding of yolk metabolism, especially of the sugars, in developing fish is still limited. Since some developing fish experience glucose production before hatching, activities for gluconeogenesis and glycogen metabolism at this stage are of interest. Recently, mass spectrometry (MS)-based measurement of metabolic activity and metabolite tracing are increasingly utilized (24): the isotope-labeled substrates administered in metabolic systems are tracked by MS. Here, using isotope tracing by MS, we describe roles of zebrafish YSL in gluconeogenesis.

## Results

### Gluconeogenesis takes place in 12–24 hpf zebrafish embryo

**Fig. 1.**
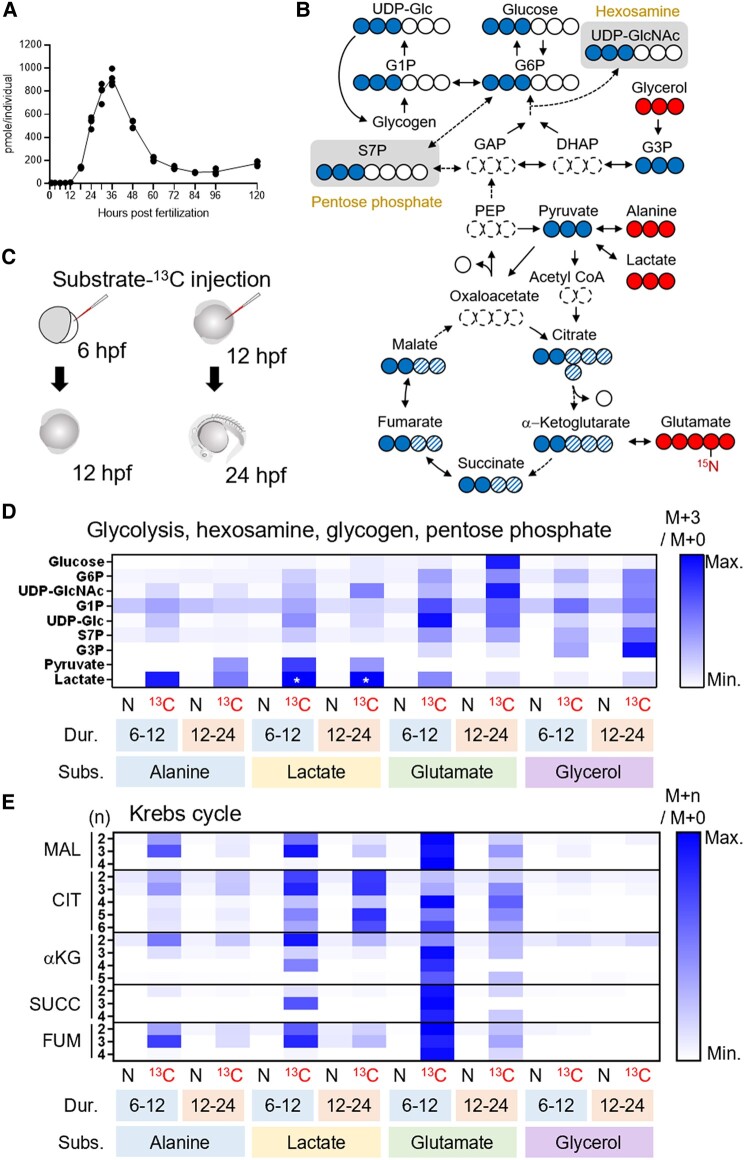
LC-MS-based isotope tracking demonstrates gluconeogenesis in zebrafish embryo. A) Glucose level increases from 12 to 36 hpf in the zebrafish. B) ^13^C-labeled isotopologues (filled circle, ^13^C; open circle, ^12^C) resulted from the substrates-^13^C (glycerol, alanine, lactate, and glutamate). For most metabolites in upper glycolysis, enrichment of mass + 3 (M + 3) isotopologues, which scarcely exist in nature, best represents the ^13^C inherited from the substrates-^13^C. Entry of substrate-^13^C into Krebs cycle gives rise to M + 2 to M + 6 isotopologues of the intermediates (filled and filled hatched circles). C) 6- or 12-hpf embryos took the injections of substrates-^13^C and given 6 or 12 h of incubation, followed by metabolite extraction. D) A rescaled heatmap showing relative M + 3 isotopologue enrichment (M + 3/M + 0) of the metabolites in upper glycolysis, hexosamine synthesis, glycogen synthesis, and pentose phosphate pathways in natural (N) or ^13^C-labeled (^13^C) substrate-injected embryos. The duration (Dur.) and substrates (Subs.) of the experiments are shown below the heatmap. Glucose M + 3 was most highly enriched in glutamate-^13^C5-injected 24-hpf embryos. Because M + 3 level of lactate was incomparably high in lactate-^13^C3-injected embryos (asterisks), these were given the same value of third-highest ones (alanine-^13^C3-injected samples) to show relative levels of other samples. E) A rescaled heatmap for relative enrichment of M + 2 to M + 5 isotopologues (M + *n*/M + 0) in Krebs cycle intermediates. Additional mass (*n*) of each isotopologue was shown on the left. αKG, α-ketoglutarate; CIT, citrate; DHAP, dihydroxyacetone phosphate; FUM, fumarate; GAP, glyceraldehyde phosphate; G1P, glucose-1-phosphate; G3P, glycerol-3-phosphate; G6P, glucose-6-phosphate; MAL, malate; PEP, phosphoenolpyruvate; SUCC, succinate; S7P, sedoheptulose-7-phosphate; UDP-Glc, UDP-glucose; UDP-GlcNAc, UDP-*N*-acetylglucosamine.

Our liquid chromatography-mass spectrometry (LC-MS) analysis found that the levels of most metabolites measured were dynamically changing in the developing zebrafish (Fig. S1), suggesting the presence of active metabolism in the embryos and larvae. Among them, glucose markedly increased from 12 hpf, peaked at 36 hpf, and then decreased to approximately 100–200 pmol/individual (Fig. 1A), which supported the result of Jurczyk et al. (11). Glycogen, the intracellularly stored polysaccharide, was also measured as the primary candidate for glucose precursor (Fig. S1). Interestingly, glycogen level also showed increasing tendency at the time of glucose surge (12–24 hpf), implying mechanisms for glucose production apart from glycogenolysis. To find out which substrate(s) contribute to the newly synthesized glucose, we employed MS-based isotope tracing technique (24). Alanine, glutamate, lactate, and glycerol were selected as possible candidates for glucose production through intermediary metabolism (Fig. 1B). These ^13^C-labeled candidate substrates were used to track the carbon flux through the metabolic pathways: these metabolites, when carried on directly to glucose, produce glucose with mass + 3 (M + 3). The ^13^C-labeled candidate substrates were injected into the yolk of 6- or 12-hpf zebrafish, which were given 6 or 12 h of incubation, respectively, followed by metabolite analysis (Fig. 1C). As a result of LC-MS-based isotopologue enrichment analysis, we found that glucose M + 3, which is most likely glucose-^13^C3, was highly enriched when the 12-hpf embryos took ^13^C-labeled glutamate and were analyzed at 24 hpf (rescaled heatmap in Figs. 1D and S2A). The result was similar when all detectable isotopologues (M + 1, M + 2, …, M + *n*) were taken into consideration, although noises from natural M + 1 and M + 2 isotopologues were reflected (Fig. S2C). These results, along with the isotopologues detected in the intermediary metabolites (Figs. 1D and E and S2C), suggest that the glutamate was metabolized through gluconeogenesis pathway: it entered Krebs cycle as α-ketoglutarate (αKG) and left this cycle via oxaloacetate (OAA)-to-phosphoenolpyruvate (PEP) rate-limiting reaction. These samples also tended to show high M + 3 enrichment in UDP-*N*-acetylglucosamine (UDP-GlcNAc), UDP-glucose, and sedoheptulose-7-phosphate (S7P), the intermediary metabolites of HBP, glycogen synthesis, and PPP, respectively. When 6-hpf embryos were supplied with ^13^C-labeled glutamate and measured at 12 hpf, glucose M + 3 level did not increase (Figs. 1D and S2A). Meanwhile, in the same embryos, M + 3 enrichment levels of glucose-6-phosphate (G6P), UDP-GlcNAc, UDP-glucose, and S7P were still much higher than natural substrate-injected controls, suggesting that “sugar production from nonsugar substrate” is occurring before glucose production initiates. Other candidate ^13^C-substrates also increased the M + 3 enrichment of glucose and other sugars measured, but the glucose M + 3 levels were modest compared to when glutamate was used as a substrate (Figs. 1D and S2A and C). Notably, during 12–24 hpf, lactate-^13^C3 robustly labeled citrate, but labeling of other Krebs-cycle metabolites was modest (Fig. 1E), implying that citrate metabolism is redirected out of Krebs cycle, or strong diluting effect of endogenous αKG derived from glutamate. We also tracked the time-course effects of glutamate-^13^C5 injection at 12 hpf (Fig. S3). Glutamate rapidly intruded into Krebs cycle, labeling αKG by 30–40% within 1 h. However, because of the dilution by natural intermediary metabolites, the levels of labeled metabolites decreased over the metabolic pathways, and the glucose labeling efficiency was 5–10% during 13–28 hpf of the experiment. Among the metabolites analyzed, glucose-1-phosphate (G1P) was only moderately labeled after ^13^C-substrate supplementation (Figs. 1D, S2C, and S3), implying active breakdown of glycogen to G1P in the embryos. Since UDP-glucose was more robustly labeled in these samples and the glycogen tended to increase between 12 and 24 hpf, the breakdown and synthesis of glycogen may occur at the same time.

### YSL expresses a set of genes for gluconeogenesis

**Fig. 2.**
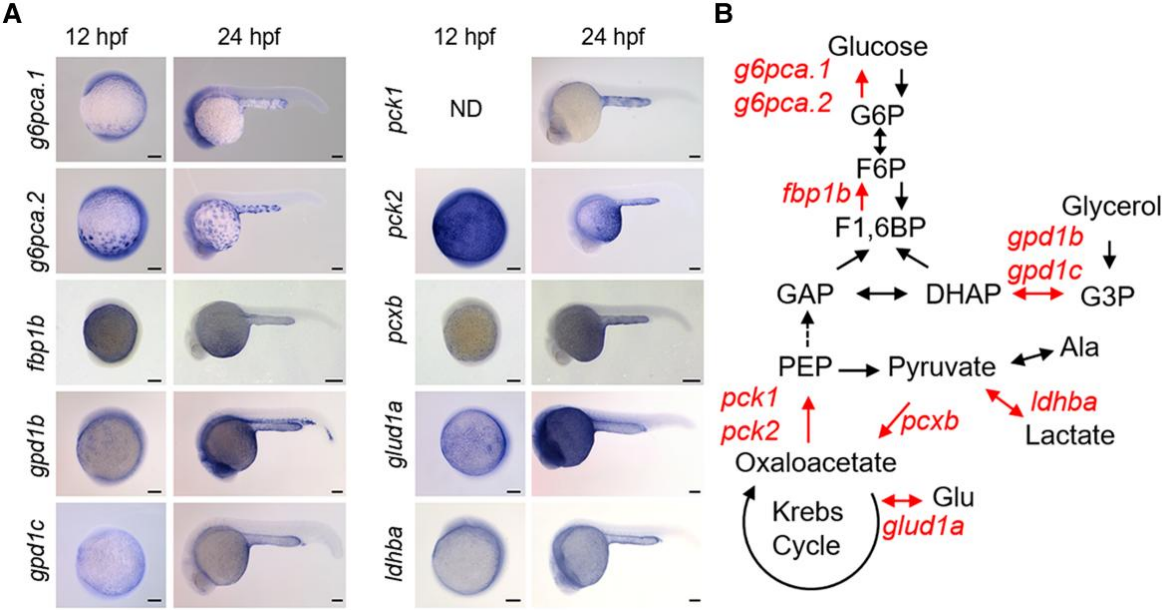
Zebrafish yolk syncytial layer (YSL) expresses genes responsible for gluconeogenesis. A) In situ hybridization signals of gluconeogenic genes expressed in YSL of zebrafish embryos at 12- and 24-h postfertilization (hpf). The gene names are shown on the left side of the panels. For sense-probe controls and genes not expressed in YSL, please see Fig. S4. Bar, 100 μm. B) The gluconeogenic genes expressed in YSL are mapped onto the pathways of gluconeogenesis. The YSL-expressed genes and the corresponding pathways were highlighted.

Like in most vertebrates, the liver and kidney are the major sites of gluconeogenesis in adult fish (25). Under 28.5°C, zebrafish liver and kidney (pronephros) start to develop at ∼30 and ∼16 hpf, respectively (13, 16). To find the tissue responsible for the embryonic gluconeogenesis, we conducted in situ hybridization (ISH) analysis and localized expression of genes encoding glucose-6-phosphatase (G6Pase), fructose-1,6-bisphosphatase (FBPase), phosphoenolpyruvate carboxykinase (PEPCK), and pyruvate carboxylase (Figs. 2 and S4), which play central roles in gluconeogenesis (for review, see Ref. (26)). Among genes assessed, *g6pca.1/g6pca.2*, *fbp1b*, *pck1/pck2*, and *pcxb* were expressed in YSL of 12- and/or 24-hpf embryos, suggesting that this tissue can carry out gluconeogenesis. Also, *gpd1b/gpd1c*, *glud1a*, and *ldhba*, encoding glycerol-3-phosphate dehydrogenase (GPD), glutamate dehydrogenase, and lactate dehydrogenase, respectively, were expressed in YSL. These enzymes are essential for gluconeogenesis using glycerol, glutamate, and lactate, as substrates. Some of these genes were quantified by qPCR analysis, and showed expression peaks before 30 hpf, the timing of liver development, suggesting active transcription of these genes in YSL (Fig. S5). These results suggest that the gluconeogenic activity found by our isotope tracing is in YSL.

### G0 knockout of *pck2* affected glucose production from glutamate

**Fig. 3.**
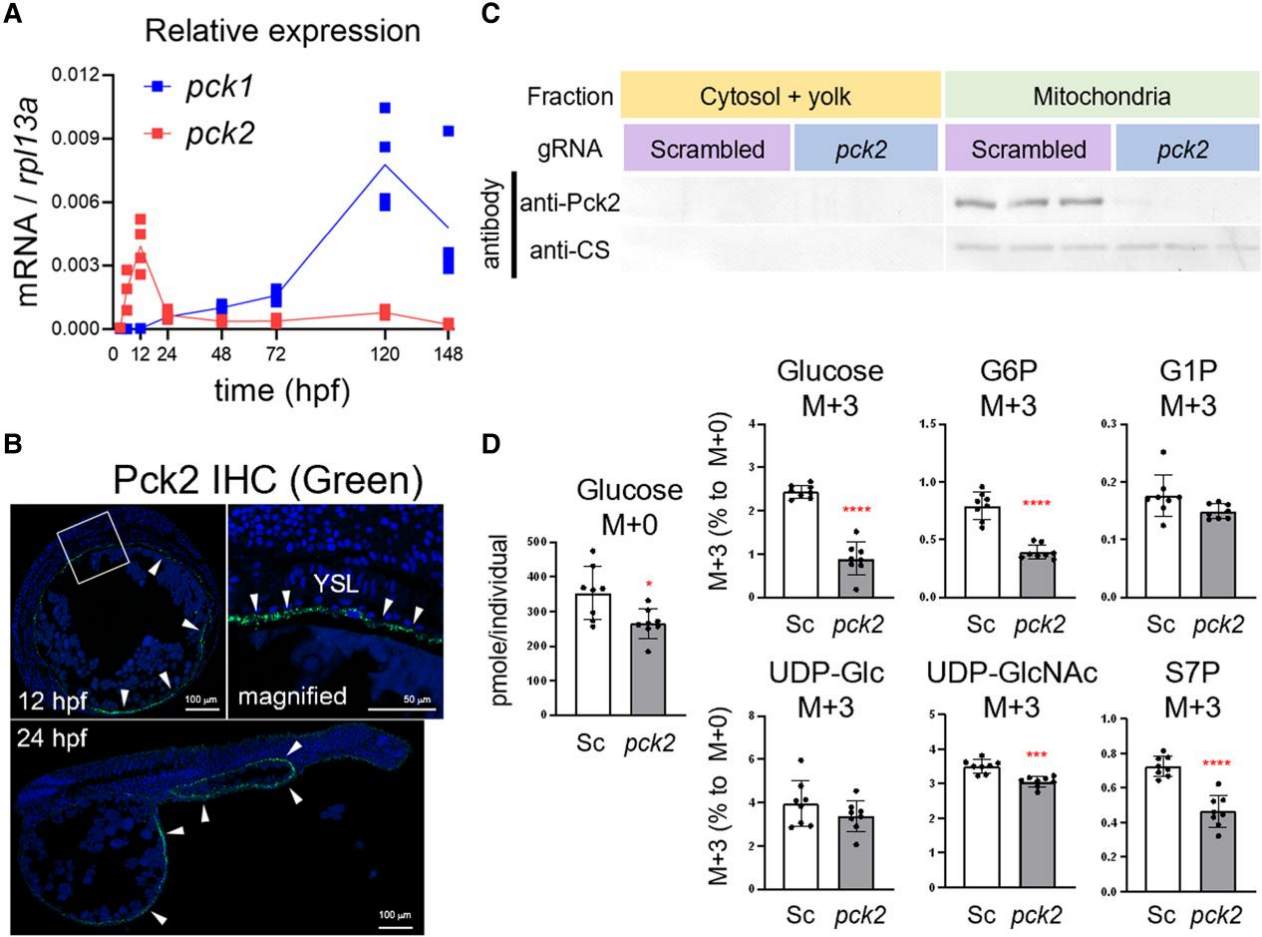
Pck2 takes a role in gluconeogenesis in YSL from glutamate. A) Relative expression levels of *pck1* and *pck2* in the developing zebrafish from 3 to 148 h postfertilization (hpf). B) Fluorescent immunohistochemistry (IHC) of the embryo sections by Pck2 antibody. Arrowheads indicate Pck2 immunosignals, and DAPI nuclei staining shows the general structure of the embryos. The squared region in the 12 hpf panel is magnified and shown on right side. Arrowheads indicate signals in YSL. (C) western blot analysis for Pck2 and citrate synthase (CS) in the 12-hpf zebrafish embryos after generation 0 (G0) knockout experiment. The fertilized eggs were injected with scrambled or *pck2*-targeting gRNA/Cas9 complex (*N* = 3). D) Levels of glucose M + 0 and relative M + 3 levels of glucose, glucose-6-phosphate (G6P), glucose-1-phosphate (G1P), UDP-glucose (UDP-Glc), UDP-*N*-acetylglucosamine (UDP-GlcNAc), and sedoheptulose-7-phosphate (S7P) in embryos subjected to G0 knockout with scrambled (Sc) or *pck2*-targeting (*pck2*) gRNA/Cas9 complex. Dots and bars denote individual and mean (±SD) values, and asterisks indicate significant difference between groups (Welch's *t* test, *P < 0.05; ***P < 0.001; ****P < 0.0001; *N* = 8).

In vertebrates, PEPCK is responsible for the essential step for gluconeogenesis from Krebs-cycle intermediates (26): it decarboxylates OAA to form PEP, while hydrolyzing GTP to GDP. There are two isoforms for this enzyme, PEPCK-C (gene: *pck1*) and PEPCK-M (*pck2*), named after their cytosolic and mitochondrial localizations, respectively (for review, see Ref. (27)). Because *pck2* is more abundantly and specifically expressed in early stages (Fig. 3A), we further assessed the role of *pck2* in the zebrafish embryo. After confirming Pck2 protein localization in zebrafish YSL by fluorescent immunohistochemistry (IHC) (Fig. 3B), we subjected the fertilized eggs to G0 knockout method (28) using *pck2*-targeting guide RNA (gRNA) and Cas9 protein. In control embryos which were treated with scrambled gRNA/Cas9 protein complex, Pck2 protein was detected by western blot analysis in mitochondrial fragment where citrate synthase was also detectable (Fig. 3C). On the other hand, the G0 knockout embryos scarcely expressed Pck2 protein. Using these embryos, the metabolic flux was assessed by injection of glutamate-^13^C5 into the yolk (Fig. 3D). In the *pck2*-targeted embryos, the levels of glucose M + 0 slightly decreased, and importantly, M + 3 enrichment levels of glucose, G6P, UDP-GlcNAc, and S7P decreased, suggesting that these metabolites, at least partially, originated from injected glutamate and *pck2* took a role in this pathway. Meanwhile, M + 3 enrichment levels of G1P and UDP-glucose, the intermediary metabolites of glycogen metabolism, were not affected by *pck2* G0 knockout. This result supports that G1P is mainly produced by breakdown of endogenous glycogen as mentioned with reference to Figs. 1D and S3. Also, YSL metabolism may be redirected to glycogen synthesis in response to *pck2* knockout because the M + 3 enrichment levels of UDP-glucose was maintained. These possibilities are further discussed in the Discussion section. Although we also tested the effects of antisense morpholino oligonucleotide (MO)-mediated knockdown or full-length cDNA-mediated forced expression of *pck2* on the metabolite flux, the results were only partially consistent with those of G0 knockout study (Fig. S6): M + 3 enrichment levels of G6P and S7P tended to decrease in *pck2* morphants and to be rescued by forced expression of *pck2*. Also, the magnitude of these changes was relatively small, and glucose M + 3 level was not changed. This discrepancy is possibly due to the mild decrease in Pck2 protein levels by MO-mediated knockdown compared to G0 knockout method. Forced expression of *pck2* tended to decrease glucose M + 0 levels, probably reflecting the effect of ectopic expression of Pck2 (Fig. S7), as cDNA overexpression takes place in widespread and mosaic manner (29). Although the effects of MO-mediated knockdown were marginal, taken together with the results of G0 knockout study, we suggest that *pck2* expressed in YSL functions as the mediator of gluconeogenic flux from glutamate.

## Discussion

Because glucose availability has profound effects on cellular homeostasis of animals, its plasma levels are maintained within narrow ranges. Gluconeogenesis by the liver and kidney is one of the central mechanisms for this function, especially during prolonged state of fast, where most liver glycogen is depleted (26). However, in early developmental stages lacking these organs, existence of such glucose-regulatory functions and their localization have been unclear. In the present study, we have clearly demonstrated the role of YSL, the extraembryonic tissue found in teleosts, in the gluconeogenesis in the developing zebrafish. Our findings shed light on a new physiological role of YSL in the developing teleost fishes.

As shown by our LC-MS metabolite tracking, all of the substrate tested, i.e. alanine, lactate, glutamate, and glycerol were utilized for glucose production in zebrafish YSL. We primarily focused on M + 3 enrichment of glucose and related metabolites because it reflects direct flux from the selected substrates but excludes their indirect effects: when labeled alanine or lactate is incorporated into Krebs cycle via acetyl-CoA, which does not contribute to replenish Krebs cycle intermediates or to gluconeogenesis, it can produce glucose M + 2 (30). Also, when labeled glutamate-derived αKG goes through one round of Krebs cycle revolution before gluconeogenesis, it results in glucose M + 2 (31). Nevertheless, we compared the results of M + 3 only (Fig. 1C) vs. all detectable isotopologue (Fig. S2C) enrichment analyses: these showed similar tendencies, supporting that glutamate-^13^C5 best labeled glucose. In the time-course study, glutamate-^13^C5 labeled glucose, marking up to 10%, even after dilution by endogenous natural metabolites. Since glutamate also labeled glutamine and aspartate (Fig. S2B), exchanges between these free amino acids are likely active in the zebrafish embryos. In this view, these free amino acids, released as the yolk protein is digested (32), are probably suitable as the substrate for gluconeogenesis. It was unexpected that glycerol did not label glucose as efficiently as glutamate did. This activity was first observed in some trouts by Terner (12). Because zebrafish embryos do not actively digest triglyceride until 24 hpf (4), the use of glycerol as substrate for gluconeogenesis is likely limited at this stage. Although our isotope tracing experiments provided important information on metabolic fluxes, the limitation of this experiment is the whole-embryo analysis due to difficulty in dissecting the embryos. Thus, careful interpretation is needed, taking possible diffusion and distribution of individual metabolite into consideration. For example, the glycerol-^13^C3 labeled G6P well but glucose only slightly (Fig. 1D). This difference implies that glycerol-^13^C3 was not only metabolized in YSL but also excreted out of yolk sac and metabolized in other tissues that express GPD but not G6Pase, such as head, tail, and hematopoietic regions (Fig. 2A). Also, the % M + 3 enrichment of G6P was less than that of glucose in embryos injected with labeled glutamate (Figs. 3D and S3). This can be explained by the dilution of G6P M + 3 with unlabeled counterpart in the embryonic tissues, where endogenous glycogen, glycerol, or other metabolites can produce G6P M + 0.

**Fig. 4.**
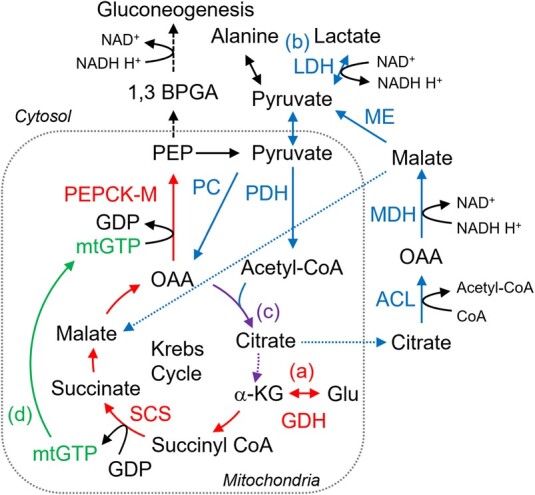
Possible metabolic pathways in YSL found in this study. The pathways originating glutamate (Glu), lactate, and both are labeled with (a), (b), and (c), respectively. Glu is converted to α-ketoglutarate (αKG) by glutamate dehydrogenase (GDH), metabolized through Krebs cycle to oxaloacetate (OAA), and used to produce phosphoenolpyruvate (PEP) via PEPCK-M reaction, while this enzyme hydrolyzes mtGTP. Here, succinyl-CoA synthetase (SCS) in Krebs cycle provides mtGTP, labeled with (d). Lactate, following conversion to pyruvate, enters Krebs cycle by two separated pathways: to OAA via pyruvate carboxylase (PC) or to acetyl-CoA via pyruvate dehydrogenase (PDH). The former provides new Krebs cycle intermediate, while the latter only helps revolution of this cycle. Considerable amount of citrate is likely transported out of mitochondria and metabolized back to pyruvate via ATP-citrate lyase (ACL), malate dehydrogenase (MDH), and malic enzyme (ME).

Our gene expression study supported the data from isotope tracking: many of the critical genes for gluconeogenesis were robustly expressed in YSL at 12 and 24 hpf. In addition, the G0 knockout study suggested the involvement of *pck2*, which encodes PEPCK-M, in the gluconeogenesis in YSL. PEPCK-M had been less studied than PEPCK-C in mammals due to its limited contribution to glycemic control (for review, see Ref. (27)). Later, however, mouse in vivo and in vitro knockdown models revealed that PEPCK-M indeed contributed to gluconeogenesis (33). This enzyme requires hydrolysis of mitochondrial GTP (mtGTP) produced by succinyl-CoA synthetase (SCS). Since mtGTP cannot be refilled from cytosolic GTP pool, mtGTP production by SCS limits the rate of PEPCK-M (27). It is reasonable that glutamate, which produces mtGTP through SCS, was a preferred substrate for gluconeogenesis via PEPCK-M in the zebrafish YSL (Fig. 4). Although alanine and lactate can circumvent Krebs cycle before reaching OAA, this “shortcut” route requires other mechanisms for mtGTP supplementation. Alternatively, these substrates may go through Krebs cycle. However, our MS-based metabolite tracking data suggest that the carbon flux from citrate to αKG is restricted in 12–24 hpf zebrafish. It is therefore possible that the citrate is prone to be metabolized by cytosolic ATP-citrate lyase (ACL) to form OAA, forming pyruvate–citrate cycle (27). This cycle, producing acetyl-CoA, may contribute to fatty acid synthesis in the embryos, as the levels of phosphatidylcholine and triglyceride showed increasing tendency until 24 hpf (4). Also, substantial labels of citrate M + 2 and citrate M + 6, detected in lactate-^13^C3-injected embryos, confirmed pyruvate dehydrogenase activity in these embryos.

To our best knowledge, gluconeogenesis in YSL had never been observed, although collective knowledge of gene expression studies (7, 11, 34) have implied existence of this phenomenon. Glucose produced in YSL may help organogenesis until the liver develops. It is interesting to know why gluconeogenesis takes place, because maternal glycogen storage, if sufficiently provided, may spare the embryos’ labor of converting nonsugar metabolites all the way to glucose. Indeed, G0 knockout of *pck2*, although accompanying partial decrease in glucose, had no effect on the survival of the embryos. Since the zebrafish embryos possess a large pool of glycogen, they could have survived the G0 knockout by mobilizing this sugar storage in YSL or the whole body for glucose production, glycolysis, or other pathways. This possibility is supported by the modest decrease in M + 0 glucose compared to M + 3 counterpart, and by the low M + 3 enrichment of G1P. Also, the lack of *pck2* G0 knockout effect on UDP-glucose M + 3 implies that the metabolic flux from the labeled glutamate in YSL was redirected to glycogen synthesis, possibly for replenishment of glycogen consumed before glutamate-^13^C injection. It is always better to have alternative choices to safely carry out important events: the intricate metabolic pathways may cooperate to cover up other limited pathways or metabolites. In addition to glycogen, glycerol and many other substrates can also contribute to glucose production without the help of PEPCK-M. Therefore, the glutamate-based gluconeogenesis found in this study may be only the part of what the zebrafish YSL is doing to meet nutritional demand of the whole animal. In nature, the quality of the eggs and the surrounding environment may vary, and the embryos’ metabolism need to respond to their own conditions and optimize the development. Also, the biological events that the embryos may face should vary among animal species, thus the importance of gluconeogenesis may depend on animal species.

Our results raise another important question: how about the other animal lineages? As mentioned, YSL and YSL-like tissues are also found in chick and sharks, but their physiological roles are still unclear. It will be interesting to compare their functional properties with those of teleost YSL. Also, if the glucose production is that important phenomenon during development, how other oviparous animals, which lack YSL, undergo this process before the formation of liver and kidney? Our findings provide important information for paving a new way to understand evolutionary developmental biology from a physiological point of view.

## Materials and methods

### Fish

Adult zebrafish (AB strain) were kept in the common laboratory of School of Marine Bioscience, Kitasato University, with dechlorinated and recirculating tap water at 28.5°C. The fertilized eggs were collected and incubated in 100-mL dishes at 29°C until the desired developmental stages were reached. All experiments were conducted in accordance with the guidelines in Kitasato University (approval nos. 4725 and 4796).

### Injection of stable isotope-labeled metabolites

L-Alanine-^13^C3 (Merck, Burlington, MA, USA), Sodium L-Lactate-^13^C3 (Cambridge Isotope Laboratories, Tewksbury, MA, USA), Glutamate-^13^C5, ^15^N (Taiyo Nippon Sanso, Tokyo, Japan), and Glycerol-^13^C3 (Taiyo Nippon Sanso) were diluted to 420 mM with deionized water. For glutamate, the pH was adjusted to 7.5 with NaOH and 100 mM HEPES. Control metabolites with natural ^13^C abundance were purchased from Fujifilm Wako Pure Chemical Corporation (Osaka, Japan), and prepared in the same manner. Two nanoliters of the prepared metabolites were injected into 6- or 12-hpf embryos.

### Metabolite analysis with LC-MS

Metabolites were extracted and analyzed with the methods previously described (35) with some modifications. 30–100 embryos or larvae were pooled in 1.5-mL tubes and anesthetized with 0.03% MS-222. After removal of excess water, 5 μL internal standard solution (l-methionine sulfone, 2-(N-morpholino) ethanesulfonic acid, d-camphor-10-sulfonic acid) and 200 μL cold methanol were added to the tubes, and the samples were homogenized with pestles. Then, 100 μL water and 200 μL chloroform were added to the samples, mixed well, and kept on ice for 10 min. The lysate was centrifuged, and aqueous phase was analyzed with the LC-MS system.

LC-MS were performed with an LC system (Prominence; Shimadzu, Kyoto, Japan) connected to a TripleTOF 5600^+^ mass spectrometer (AB Sciex, Framingham, MA, USA). Four HILIC methods were used with Shodex HILICpak VT-50 2D (Showa Denko, Tokyo, Japan) and Shodex HILICpak VG-50 2D (Showa Denko) columns. The detailed methods and the metabolites measured were listed in Tables S1 and S2. The samples were run in parallel with standards with known concentrations, and the analyte levels were quantified with MultiQuant software (AB Sciex). The levels of metabolites were normalized by those of internal standards. Glycogen levels were expressed as those of glucose increased after glucoamylase digestion.

### Isotopologue enrichment analysis

The mass + *n* (M + *n*) isotopologues were detected at *m*/*z* of additional 1.00325 * *n* and at the same retention time as mass + 0 (M + 0) isotopologues. The relative M + *n* enrichment was expressed as peak areas of (M + *n*)/(M + 0). Relative enrichment of the isotopologues in samples was rescaled from 0 (lowest) to 1 (highest), to better represent differences in M + *n* abundance among the groups. For all metabolites measured, the standard curves were drawn with natural metabolites to check ranges of linear relationship between concentration and MS signals. Known concentrations of standard chemicals were injected and the lower limit of quantitation was determined by signals of repeated injections of blank controls.

### qPCR and whole-mount ISH analysis

The total RNA was extracted from developing zebrafish at 3, 6, 12, 24, 48, 72, 120, and 148 hpf with TRIzol (Thermo Fisher Scientific, Waltham, MA, USA), digested with DNase I, and reverse-transcribed with SuperScript III (Thermo Fisher Scientific). qPCR was performed with LightCycler 480 system II (Roche Diagnostics, Mannheim, Germany) and LightCycler FastStart DNA Master^PLUS^ SYBR Green I (Roche Diagnostics). The primers used are listed in Table S3. The levels of transcripts were quantified by comparing to parallel amplifications of serial dilutions of standard plasmids and then normalized with the levels of *rpl13a*.

Whole-mount ISH was performed as reported by Thisse and Thisse (36). Briefly, the RNA probes were produced by in vitro transcription reaction in the presence of T7 or SP6 polymerase, DIG Labeling Mix (Roche Diagnostics) and target cDNAs in pGEM T-Easy Vector (Promega, Madison, WI, USA). The 12- or 24-hpf zebrafish embryos were fixed in 4% paraformaldehyde (PFA) in phosphate-buffered saline prepared in diethylpyrocarbonate-treated water at 4°C overnight, and then kept in 100% methanol at −20°C. In 48-well plates, the embryos were rehydrated with PBS containing 0.1% Tween 20 (PBST)-DEPC, digested with proteinase K (Roche Diagnostics), and hybridized in 300 μL of HM^+^ buffer (Thisse and Thisse) containing 100 ng of antisense or sense (negative control) probes. After washing, the hybridized probes were visualized with NBT/BCIP reaction, and the pictures were taken with a Z16 APO microscope (Leica Microsystems, Heerbrugg, Germany) equipped with a Leica DFC420C digital camera. For *fbp1a*, *fbp1b*, *pcxa*, and *pcxb*, the images were obtained by SZX10 (Olympus, Tokyo, Japan) with FX380 digital camera. For the enzymes with multiple homologs, candidate genes were selected by RT-PCR analysis for robust expression or by referencing zfin gene expression database (34): *g6pc*3 and *fbp2* were excluded because of very low expression levels, and *ldha*, *ldhbb*, and *fah* were excluded because their expression in muscle or liver, but not in YSL, is clearly shown in zfin database.

### G0 knockout, MO-mediated knockdown, and forced expression of *pck2*

Four different scrambled gRNAs without a targeting sequence and specific gRNAs targeting four sites of zebrafish *pck2* were synthesized according to Wu et al. (28), with the scaffold, scrambled, and *pck2* primers (Table S3). The gRNA mix (1 μg/μL) and Cas9 NLS (1.5 μg/μL) were mixed 1:1 and assembled at 37°C for 10, and 2 nL of this gRNA/Cas9 complex solution was injected into the fertilized zebrafish eggs. For MO-mediated knockdown study, a specific MO designed for zebrafish *pck2* (sequence; TGGAGCAAACAAGATATTGTGGCTT) was purchased from Gene Tools (Philomath, OR). The MO was diluted to 2 ng/nL, and 2 ng MO was injected into the yolk of the fertilized eggs. For the rescue and overexpression experiments, the full-length *pck2* cDNA was cloned into pCS2+ vector, linearized with *Hpa* I, and 90 pg of the DNA was injected in combination with the MO.

The efficiency of the G0 knockout, MO-mediated knockdown, and forced expression of *pck2* was confirmed by western blot analysis. Antizebrafish Pck2 polyclonal antibody was raised in mouse against the C-terminal 87-amino acid peptide of Pck2 and affinity-purified (Protein Purify, Gunma, Japan). One hundred zebrafish embryos were pooled, and mitochondrial protein was extracted according to Prudent et al. (37). Briefly, 100 embryos were pooled in a tube, homogenized in MB buffer with loose-fitting pestles, and centrifuged at 1,500 × *g* for 10 min. Then, the supernatant was further centrifuged at 10,600 × *g* for 10 min to pellet mitochondria. The mitochondrial fraction was washed with MB buffer and reconstituted in 20 μL of the same buffer with 1% sodium deoxycholate. The supernatant from the second centrifugation was used as cytosolic fraction. Ten-microgram protein was subjected to western blot analysis with the antizebrafish Pck2 antibody. The signals obtained by anticitrate synthase antibody (GTX628143; Gene Tex, Irvine, CA, USA) was used as relative levels of mitochondrial protein.

### Fluorescent IHC

The 12- and 24-hpf zebrafish embryos were dechorionated, fixed in 4% PFA overnight at 4°C, and stored in 70% ethanol. The samples were dehydrated, embedded in paraffin, and sectioned in 5-μm thickness. The sections were affixed to glass slides, deparaffinated, blocked in 1×Western Blocking Reagent (Roche Diagnostics), and incubated in anti-Pck2 antibody diluted 1:2,000 in the blocking buffer overnight at 4°C. Next day, the sections were washed and further incubated in Alexa Fluor 488-conjugated antimouse IgG (ab150113; Abcam, Cambridge, UK) diluted 1:1,000 in PBS. The sections were stained with DAPI (Dojindo Laboratories, Kumamoto, Japan) diluted 1:1,000 in PBS, mounted in Vectashield mounting medium (H-1000; Vector Laboratories, Burlingame, CA, USA), and photographed with LSM800 confocal microscope (Carl Zeiss, Oberkochen, Germany).

## Supplementary Material

pgae125_Supplementary_Data

## Data Availability

All data are included in the manuscript and supporting information.
