# Supplementary material for: Gluconeogenesis in the extraembryonic yolk syncytial layer of the zebrafish embryo
Source: PNAS Nexus. 2024 Mar 21;3(4):pgae125. doi: 10.1093/pnasnexus/pgae125 (PMC10997050; doi:10.1093/pnasnexus/pgae125)
Supplement: pgae125_Supplementary_Data [file pgae125_supplementary_data.zip › PNASNEXUS-PNASNEXUS-2023-00554R-s01.pptx]

## Slide 1
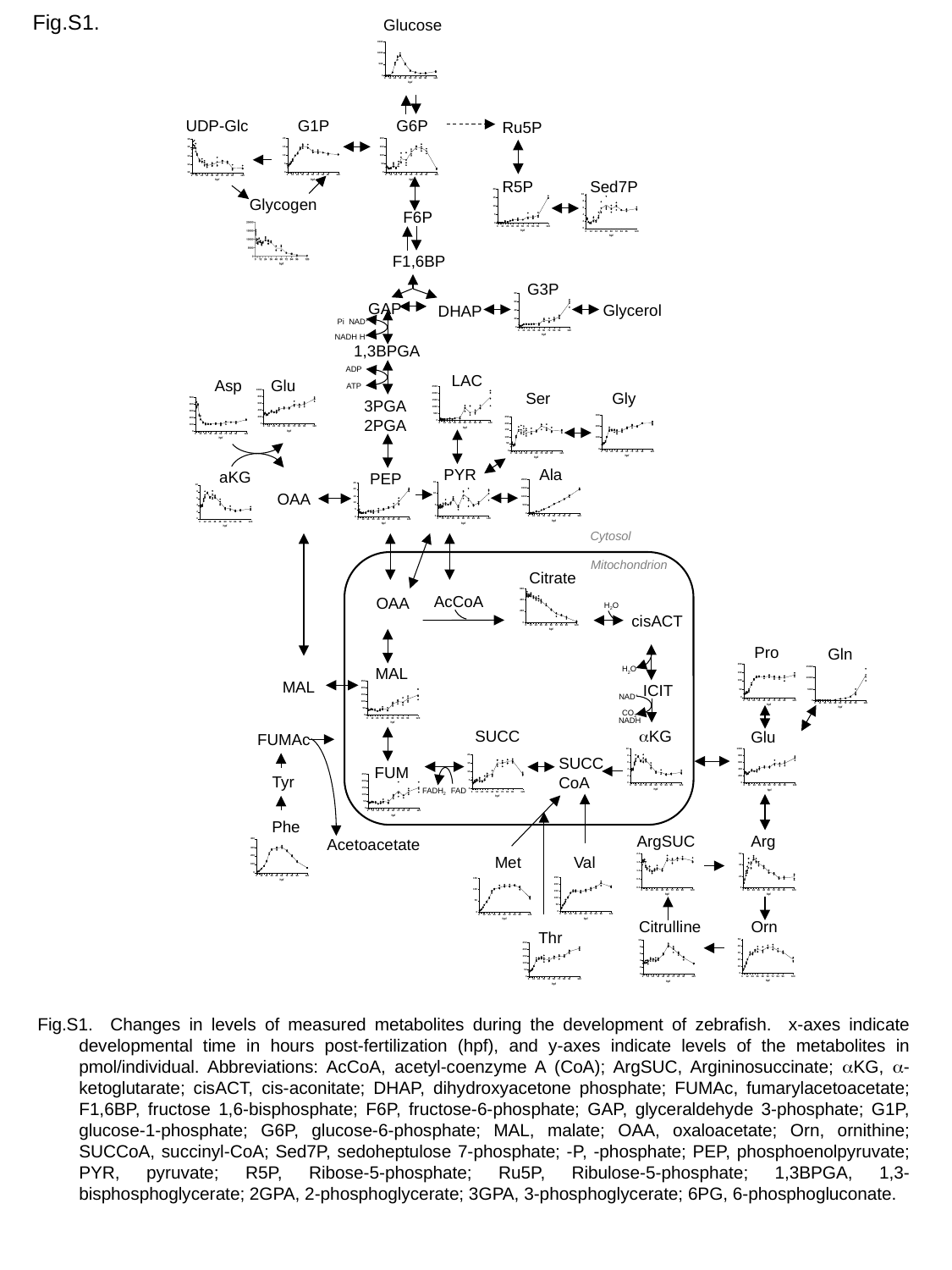

Fig.S1.
Glucose
UDP-Glc
G1P
G6P
Ru5P
Sed7P
R5P
Glycogen
F6P
F1,6BP
G3P
GAP
Glycerol
DHAP
Pi NAD+
NADH H+
1,3BPGA
ADP
LAC
Asp
Glu
ATP
Ser
Gly
3PGA
2PGA
PYR
Ala
aKG
PEP
OAA
Cytosol
Mitochondrion
Citrate
AcCoA
OAA
H2O
cisACT
Pro
Gln
MAL
H2O
MAL
ICIT
NAD+
CO2
NADH
aKG
SUCC
Glu
FUMAc
SUCC
CoA
FUM
Tyr
FADH2
FAD
Phe
ArgSUC
Arg
Acetoacetate
Met
Val
Citrulline
Orn
Thr
 Fig.S1. Changes in levels of measured metabolites during the development of zebrafish. x-axes indicate developmental time in hours post-fertilization (hpf), and y-axes indicate levels of the metabolites in pmol/individual. Abbreviations: AcCoA, acetyl-coenzyme A (CoA); ArgSUC, Argininosuccinate; aKG, a-ketoglutarate; cisACT, cis-aconitate; DHAP, dihydroxyacetone phosphate; FUMAc, fumarylacetoacetate; F1,6BP, fructose 1,6-bisphosphate; F6P, fructose-6-phosphate; GAP, glyceraldehyde 3-phosphate; G1P, glucose-1-phosphate; G6P, glucose-6-phosphate; MAL, malate; OAA, oxaloacetate; Orn, ornithine; SUCCoA, succinyl-CoA; Sed7P, sedoheptulose 7-phosphate; -P, -phosphate; PEP, phosphoenolpyruvate; PYR, pyruvate; R5P, Ribose-5-phosphate; Ru5P, Ribulose-5-phosphate; 1,3BPGA, 1,3-bisphosphoglycerate; 2GPA, 2-phosphoglycerate; 3GPA, 3-phosphoglycerate; 6PG, 6-phosphogluconate.
