# Supplementary material for: Gluconeogenesis in the extraembryonic yolk syncytial layer of the zebrafish embryo
Source: PNAS Nexus. 2024 Mar 21;3(4):pgae125. doi: 10.1093/pnasnexus/pgae125 (PMC10997050; doi:10.1093/pnasnexus/pgae125)
Supplement: pgae125_Supplementary_Data [file pgae125_supplementary_data.zip › PNASNEXUS-PNASNEXUS-2023-00554R-s02.pptx]

## Slide 1
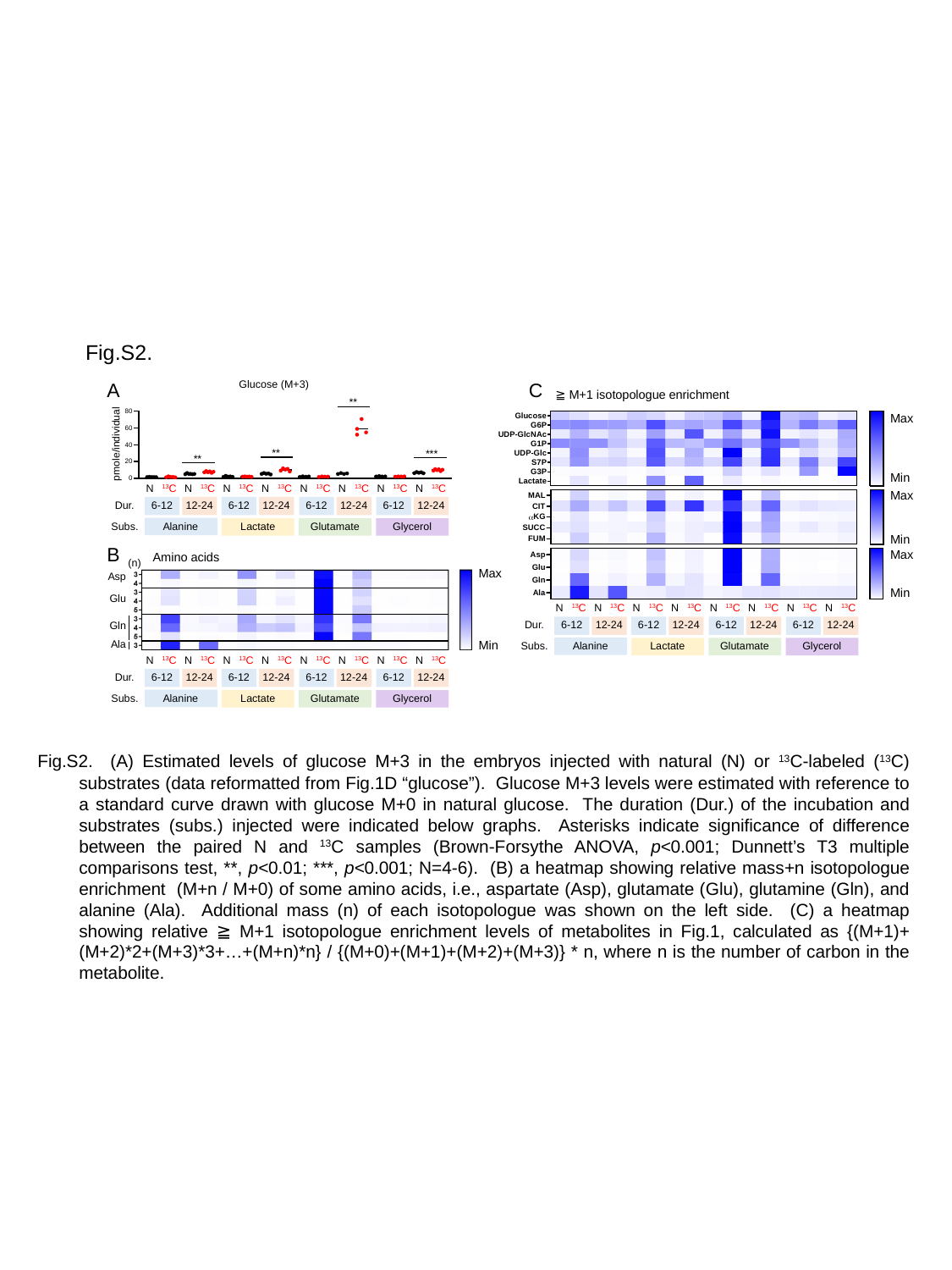

Fig.S2.
Glucose (M+3)
A
C
≧ M+1 isotopologue enrichment
**
Max
**
***
**
Min
N
13C
N
13C
N
13C
N
13C
N
13C
N
13C
N
13C
N
13C
Max
Dur.
6-12
12-24
6-12
12-24
6-12
12-24
6-12
12-24
Subs.
Alanine
Lactate
Glutamate
Glycerol
Min
B
Max
Amino acids
(n)
Max
Asp
Min
Glu
N
13C
N
13C
N
13C
N
13C
N
13C
N
13C
N
13C
N
13C
Dur.
6-12
12-24
6-12
12-24
6-12
12-24
6-12
12-24
Gln
Ala
Min
Subs.
Alanine
Lactate
Glutamate
Glycerol
N
13C
N
13C
N
13C
N
13C
N
13C
N
13C
N
13C
N
13C
Dur.
6-12
12-24
6-12
12-24
6-12
12-24
6-12
12-24
Subs.
Alanine
Lactate
Glutamate
Glycerol
 Fig.S2. (A) Estimated levels of glucose M+3 in the embryos injected with natural (N) or 13C-labeled (13C) substrates (data reformatted from Fig.1D “glucose”). Glucose M+3 levels were estimated with reference to a standard curve drawn with glucose M+0 in natural glucose. The duration (Dur.) of the incubation and substrates (subs.) injected were indicated below graphs. Asterisks indicate significance of difference between the paired N and 13C samples (Brown-Forsythe ANOVA, p<0.001; Dunnett’s T3 multiple comparisons test, **, p<0.01; ***, p<0.001; N=4-6). (B) a heatmap showing relative mass+n isotopologue enrichment (M+n / M+0) of some amino acids, i.e., aspartate (Asp), glutamate (Glu), glutamine (Gln), and alanine (Ala). Additional mass (n) of each isotopologue was shown on the left side. (C) a heatmap showing relative ≧ M+1 isotopologue enrichment levels of metabolites in Fig.1, calculated as {(M+1)+(M+2)*2+(M+3)*3+…+(M+n)*n} / {(M+0)+(M+1)+(M+2)+(M+3)} * n, where n is the number of carbon in the metabolite.
