# Supplementary material for: Gluconeogenesis in the extraembryonic yolk syncytial layer of the zebrafish embryo
Source: PNAS Nexus. 2024 Mar 21;3(4):pgae125. doi: 10.1093/pnasnexus/pgae125 (PMC10997050; doi:10.1093/pnasnexus/pgae125)
Supplement: pgae125_Supplementary_Data [file pgae125_supplementary_data.zip › PNASNEXUS-PNASNEXUS-2023-00554R-s03.pptx]

## Slide 1
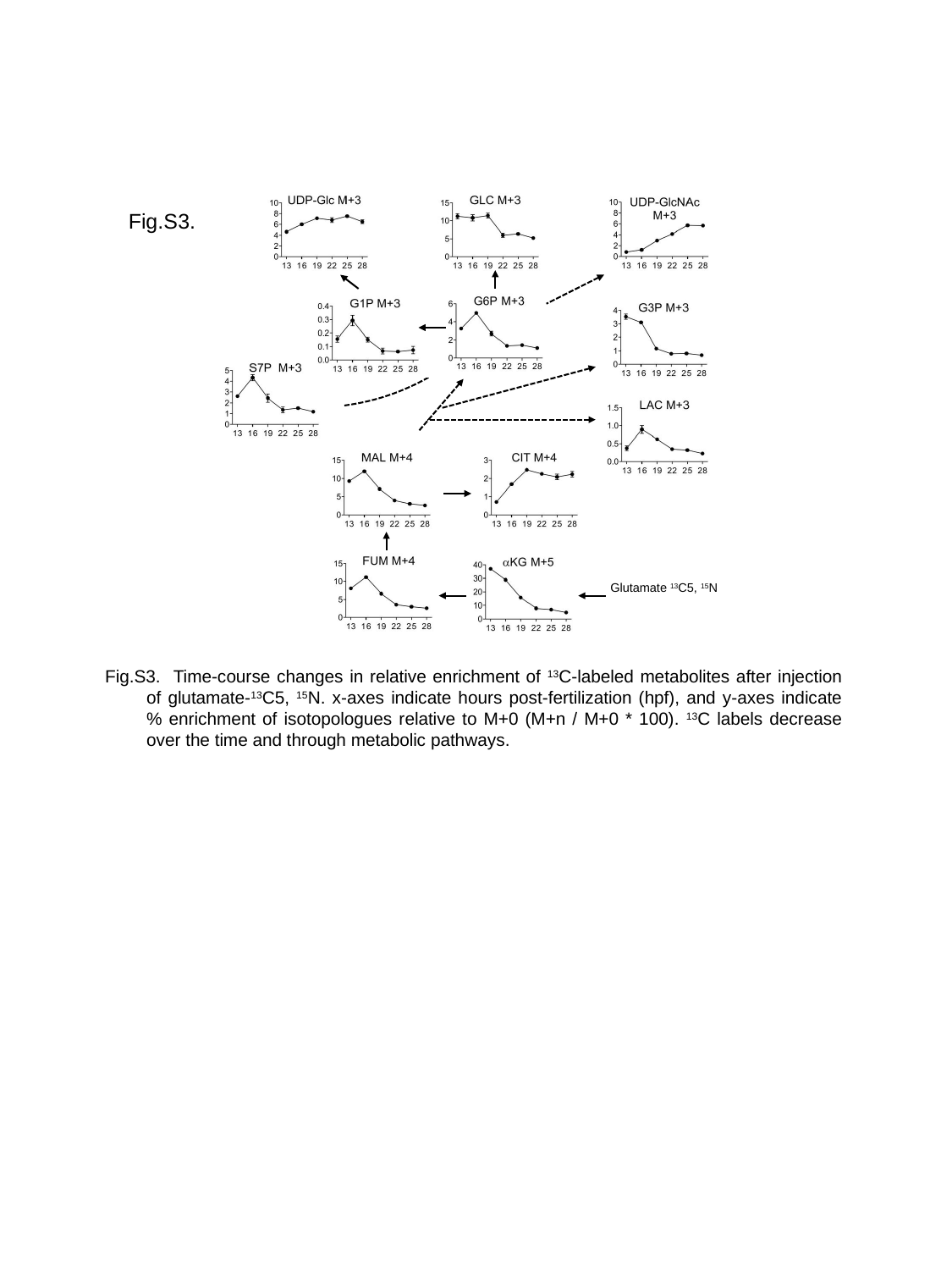

Glutamate 13C5, 15N
Fig.S3.
 Fig.S3. Time-course changes in relative enrichment of 13C-labeled metabolites after injection of glutamate-13C5, 15N. x-axes indicate hours post-fertilization (hpf), and y-axes indicate % enrichment of isotopologues relative to M+0 (M+n / M+0 * 100). 13C labels decrease over the time and through metabolic pathways.
