# Supplementary material for: Gluconeogenesis in the extraembryonic yolk syncytial layer of the zebrafish embryo
Source: PNAS Nexus. 2024 Mar 21;3(4):pgae125. doi: 10.1093/pnasnexus/pgae125 (PMC10997050; doi:10.1093/pnasnexus/pgae125)
Supplement: pgae125_Supplementary_Data [file pgae125_supplementary_data.zip › PNASNEXUS-PNASNEXUS-2023-00554R-s04.pptx]

## Slide 1
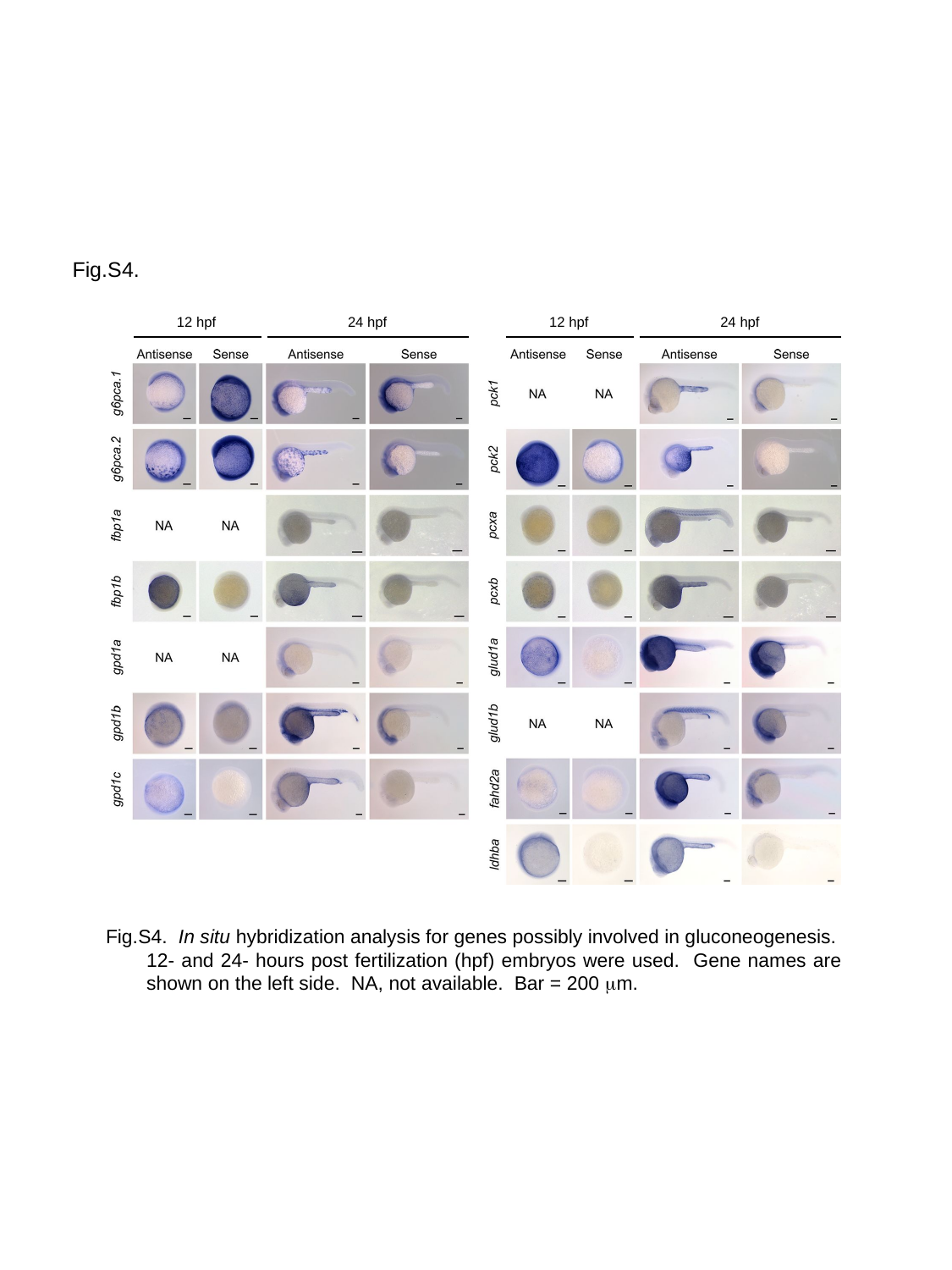

Fig.S4.
12 hpf
24 hpf
12 hpf
24 hpf
 Fig.S4. In situ hybridization analysis for genes possibly involved in gluconeogenesis. 12- and 24- hours post fertilization (hpf) embryos were used. Gene names are shown on the left side. NA, not available. Bar = 200 mm.
