# Supplementary material for: Gluconeogenesis in the extraembryonic yolk syncytial layer of the zebrafish embryo
Source: PNAS Nexus. 2024 Mar 21;3(4):pgae125. doi: 10.1093/pnasnexus/pgae125 (PMC10997050; doi:10.1093/pnasnexus/pgae125)
Supplement: pgae125_Supplementary_Data [file pgae125_supplementary_data.zip › PNASNEXUS-PNASNEXUS-2023-00554R-s05.pptx]

## Slide 1
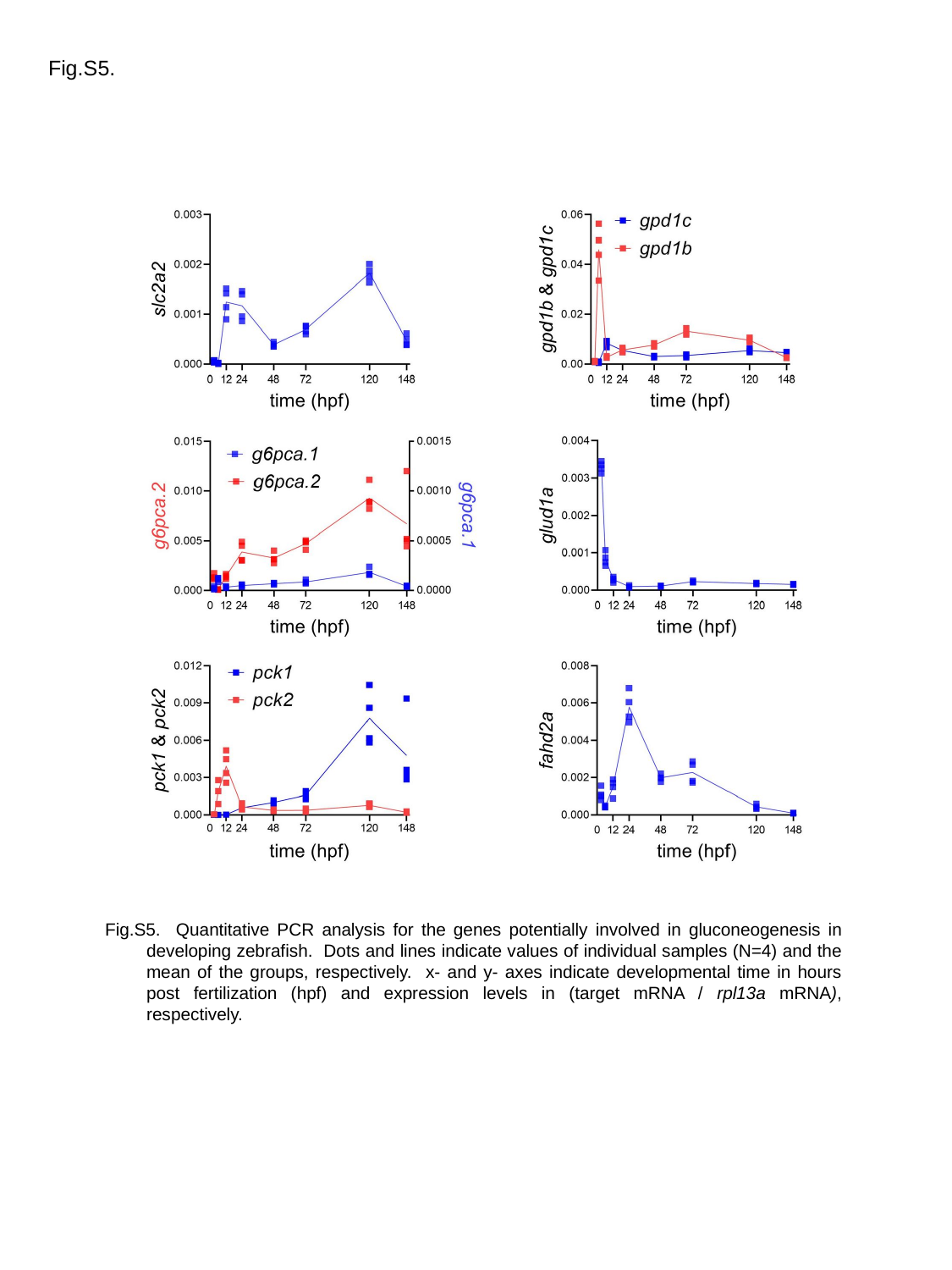

Fig.S5.
 Fig.S5. Quantitative PCR analysis for the genes potentially involved in gluconeogenesis in developing zebrafish. Dots and lines indicate values of individual samples (N=4) and the mean of the groups, respectively. x- and y- axes indicate developmental time in hours post fertilization (hpf) and expression levels in (target mRNA / rpl13a mRNA), respectively.
