# Supplementary material for: Gluconeogenesis in the extraembryonic yolk syncytial layer of the zebrafish embryo
Source: PNAS Nexus. 2024 Mar 21;3(4):pgae125. doi: 10.1093/pnasnexus/pgae125 (PMC10997050; doi:10.1093/pnasnexus/pgae125)
Supplement: pgae125_Supplementary_Data [file pgae125_supplementary_data.zip › PNASNEXUS-PNASNEXUS-2023-00554R-s06.pptx]

## Slide 1
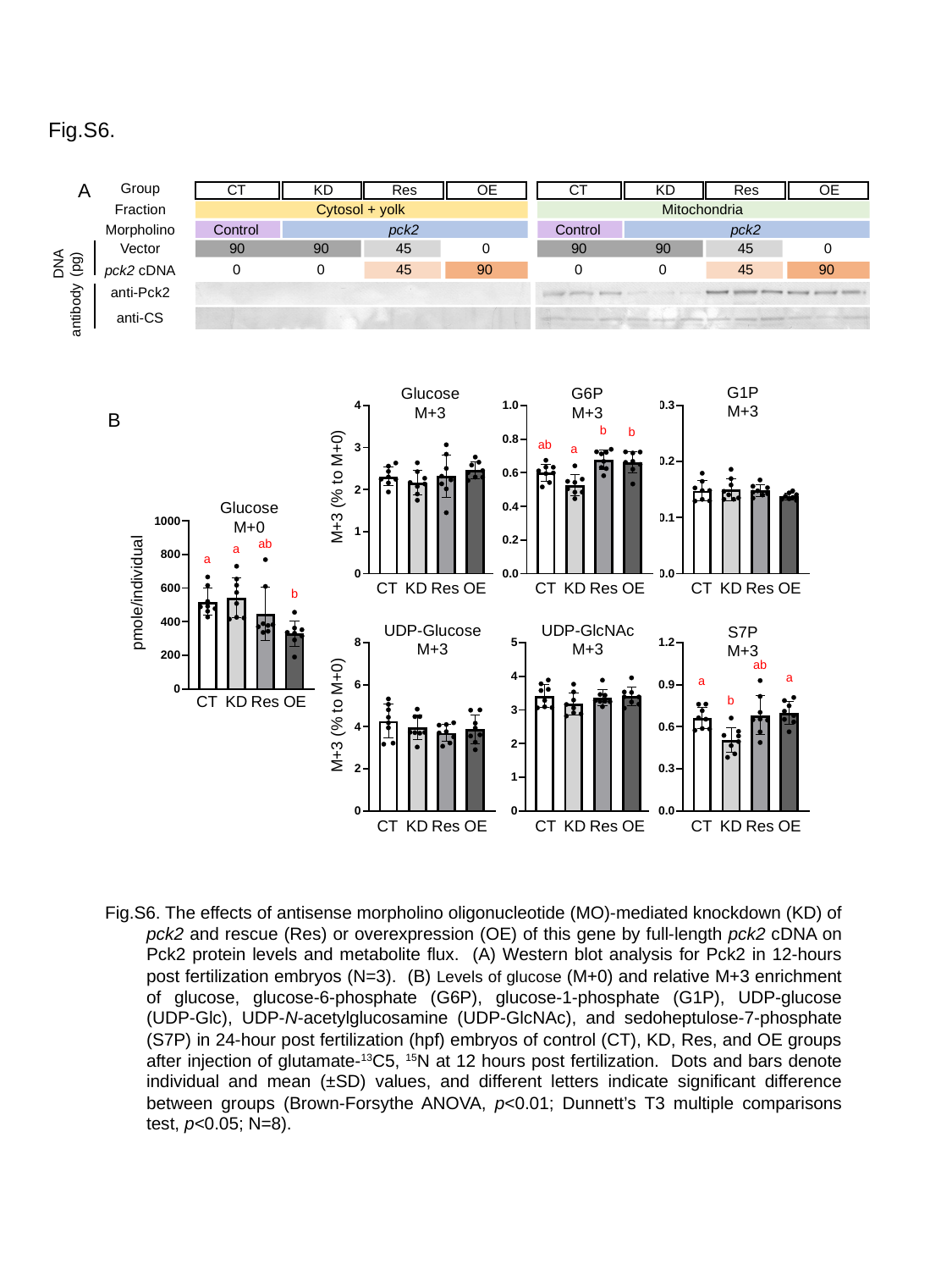

Fig.S6.
A
Group
CT
KD
Res
OE
CT
KD
Res
OE
Cytosol + yolk
Mitochondria
Fraction
Morpholino
Control
pck2
Control
pck2
0
0
90
90
45
90
90
45
Vector
DNA
(pg)
90
90
0
0
45
0
0
45
pck2 cDNA
anti-Pck2
antibody
anti-CS
G1P
M+3
Glucose
M+3
G6P
M+3
B
b
b
ab
a
M+3 (% to M+0)
Glucose
M+0
ab
a
a
CT
KD
Res
OE
CT
KD
Res
OE
CT
KD
Res
OE
pmole/individual
b
UDP-Glucose
M+3
UDP-GlcNAc
M+3
S7P
M+3
ab
a
a
CT
KD
Res
OE
b
M+3 (% to M+0)
CT
KD
Res
OE
CT
KD
Res
OE
CT
KD
Res
OE
 Fig.S6. The effects of antisense morpholino oligonucleotide (MO)-mediated knockdown (KD) of pck2 and rescue (Res) or overexpression (OE) of this gene by full-length pck2 cDNA on Pck2 protein levels and metabolite flux. (A) Western blot analysis for Pck2 in 12-hours post fertilization embryos (N=3). (B) Levels of glucose (M+0) and relative M+3 enrichment of glucose, glucose-6-phosphate (G6P), glucose-1-phosphate (G1P), UDP-glucose (UDP-Glc), UDP-N-acetylglucosamine (UDP-GlcNAc), and sedoheptulose-7-phosphate (S7P) in 24-hour post fertilization (hpf) embryos of control (CT), KD, Res, and OE groups after injection of glutamate-13C5, 15N at 12 hours post fertilization. Dots and bars denote individual and mean (±SD) values, and different letters indicate significant difference between groups (Brown-Forsythe ANOVA, p<0.01; Dunnett’s T3 multiple comparisons test, p<0.05; N=8).
