# Supplementary material for: Gluconeogenesis in the extraembryonic yolk syncytial layer of the zebrafish embryo
Source: PNAS Nexus. 2024 Mar 21;3(4):pgae125. doi: 10.1093/pnasnexus/pgae125 (PMC10997050; doi:10.1093/pnasnexus/pgae125)
Supplement: pgae125_Supplementary_Data [file pgae125_supplementary_data.zip › PNASNEXUS-PNASNEXUS-2023-00554R-s07.pptx]

## Slide 1
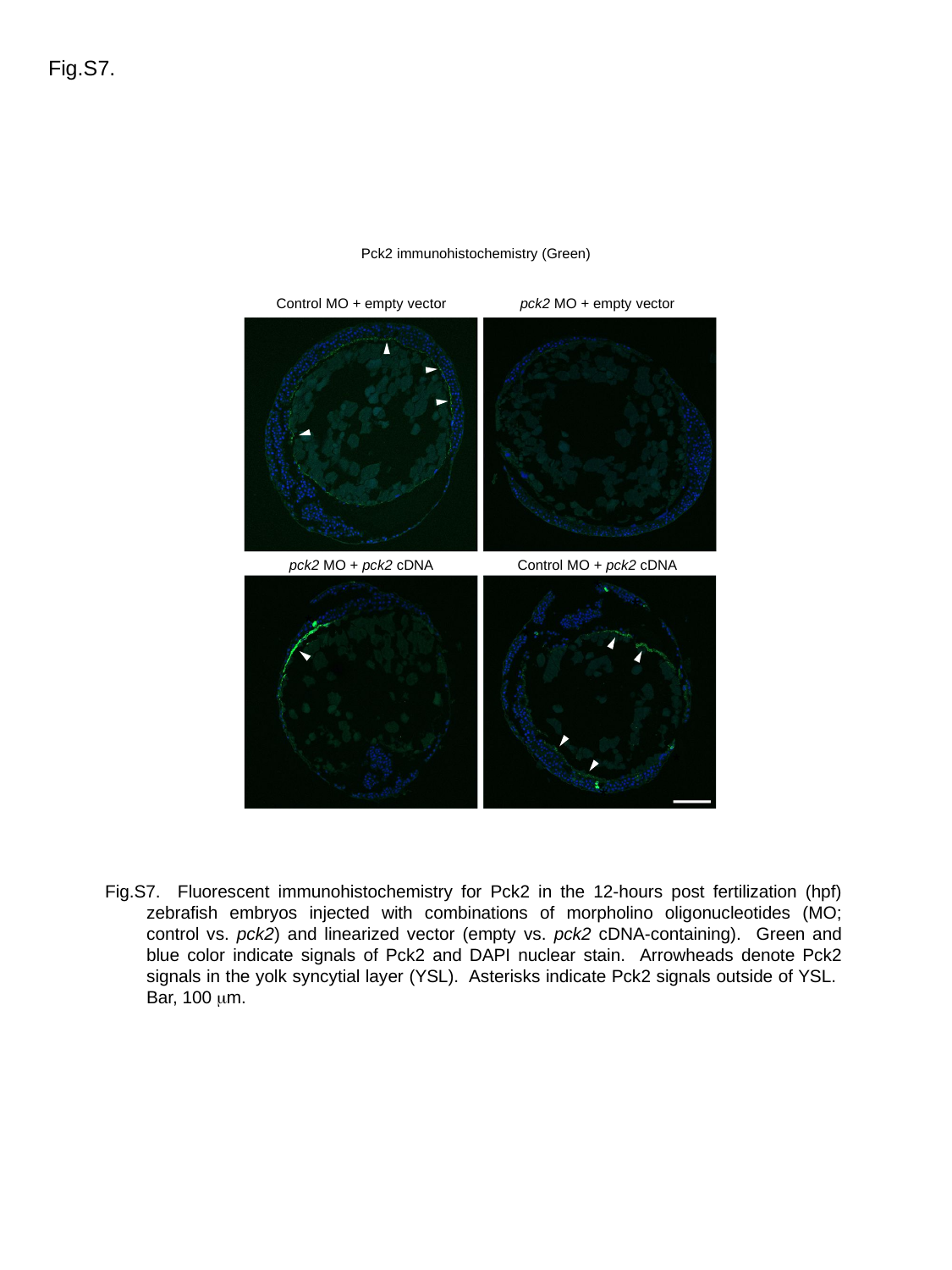

Fig.S7.
Pck2 immunohistochemistry (Green)
Control MO + empty vector
pck2 MO + empty vector
pck2 MO + pck2 cDNA
Control MO + pck2 cDNA
*
*
*
*
*
*
 Fig.S7. Fluorescent immunohistochemistry for Pck2 in the 12-hours post fertilization (hpf) zebrafish embryos injected with combinations of morpholino oligonucleotides (MO; control vs. pck2) and linearized vector (empty vs. pck2 cDNA-containing). Green and blue color indicate signals of Pck2 and DAPI nuclear stain. Arrowheads denote Pck2 signals in the yolk syncytial layer (YSL). Asterisks indicate Pck2 signals outside of YSL. Bar, 100 mm.
